# Supplementary material for: Identification of Novel Mitochondrial Pyruvate Carrier Inhibitors by Homology Modeling and Pharmacophore-Based Virtual Screening
Source: Biomedicines. 2022 Feb 2;10(2):365. doi: 10.3390/biomedicines10020365 (PMC8962382; doi:10.3390/biomedicines10020365)

Full blot images for Figure 3

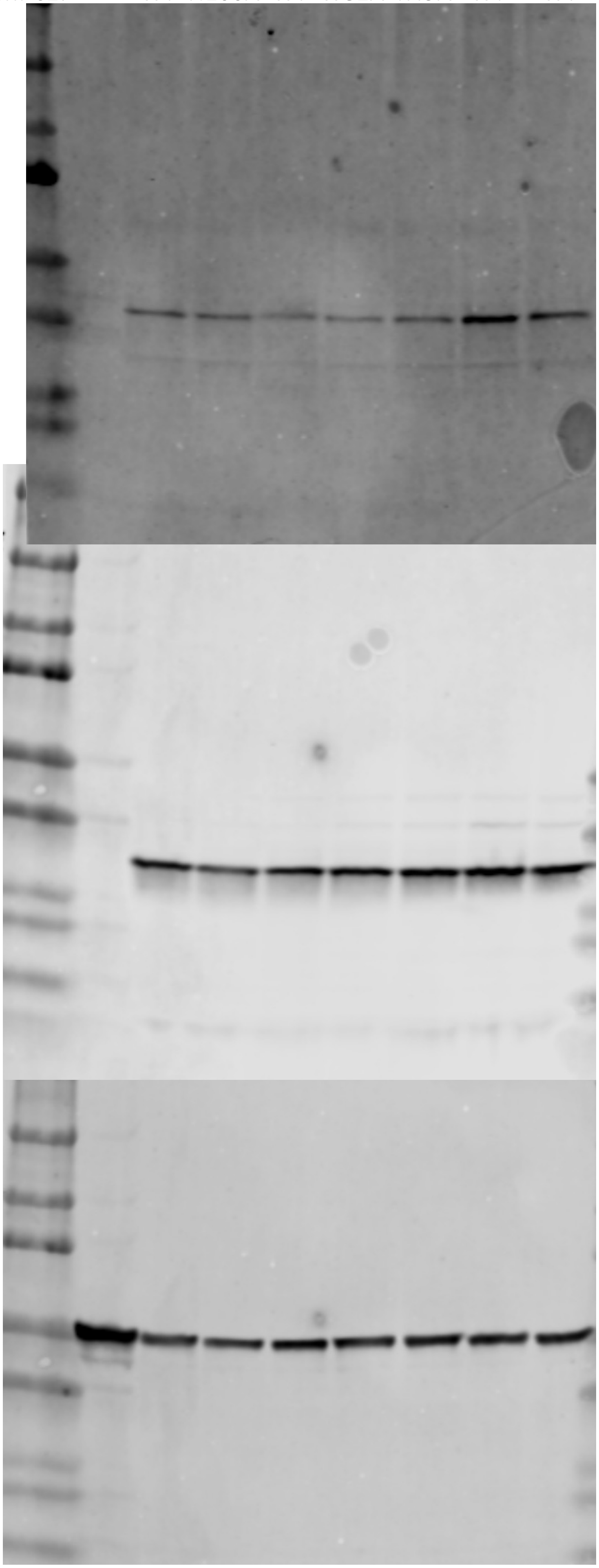

Full blot images for Figure 3

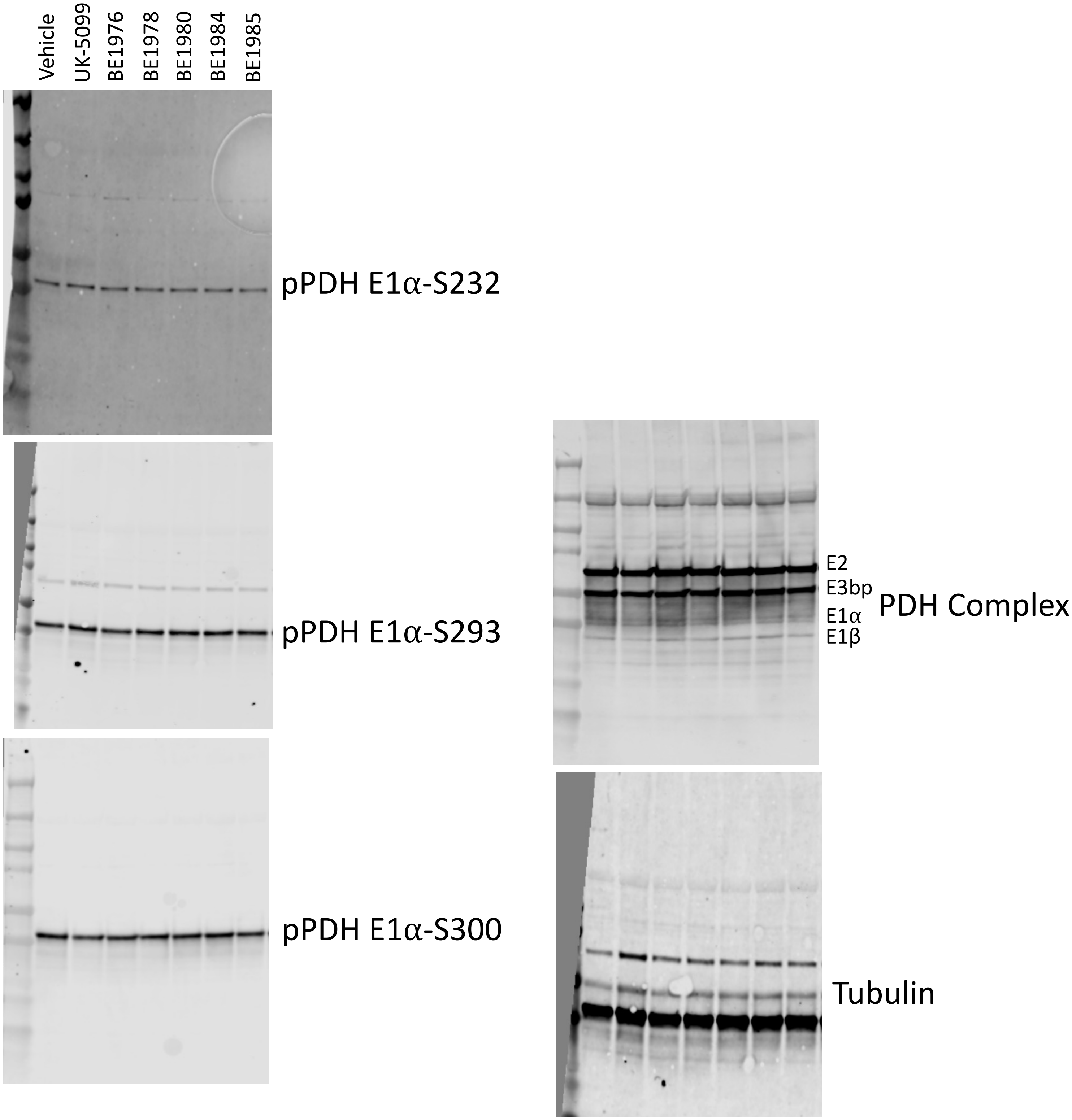

Supplement: Supplementary file 1 [file biomedicines-10-00365-s001.zip › biomedicines-1547097-supplementary.pdf]
